# Mitigating data quality challenges in ambulatory wrist-worn wearable monitoring through analytical and practical approaches

**DOI:** 10.1038/s41598-024-67767-3

**Published:** 2024-07-30

**Authors:** Jonas Van Der Donckt, Nicolas Vandenbussche, Jeroen Van Der Donckt, Stephanie Chen, Marija Stojchevska, Mathias De Brouwer, Bram Steenwinckel, Koen Paemeleire, Femke Ongenae, Sofie Van Hoecke

**Affiliations:** 1 IDLab, Ghent University - Imec, Technologiepark-Zwijnaarde, 9052 Ghent, Belgium; 2 Department of Neurology, Ghent University Hospital, Corneel Heymanslaan 10, 9000 Ghent, Belgium; 3 Department of Basic and Applied Medical Sciences, Faculty of Medicine and Health Sciences, Ghent University, Ghent, Belgium

**Keywords:** Translational research, Computer science

## Abstract

Chronic disease management and follow-up are vital for realizing sustained patient well-being and optimal health outcomes. Recent advancements in wearable technologies, particularly wrist-worn devices, offer promising solutions for longitudinal patient monitoring, replacing subjective, intermittent self-reporting with objective, continuous monitoring. However, collecting and analyzing data from wearables presents several challenges, such as data entry errors, non-wear periods, missing data, and wearable artifacts. In this work, we explore these data analysis challenges using two real-world datasets (mBrain21 and ETRI lifelog2020). We introduce practical countermeasures, including participant compliance visualizations, interaction-triggered questionnaires to assess personal bias, and an optimized pipeline for detecting non-wear periods. Additionally, we propose a visualization-oriented approach to validate processing pipelines using scalable tools such as tsflex and Plotly-Resampler. Lastly, we present a bootstrapping methodology to evaluate the variability of wearable-derived features in the presence of partially missing data segments. Prioritizing transparency and reproducibility, we provide open access to our detailed code examples, facilitating adaptation in future wearable research. In conclusion, our contributions provide actionable approaches for improving wearable data collection and analysis.

## Introduction

In recent years, wearable sensing has seen a vast increase in both research and commercialization, driven by the reduced networking and hardware costs as well as the non-intrusive nature of these devices^1^. Wearable technologies offer promising solutions for patient monitoring by continuously acquiring objective physiological data unobtrusively and at scale. As such, wearable sensing could potentially ease the strain on the healthcare system, particularly in managing chronic diseases^2,3^. For instance, diabetes patients could benefit from real-time tracking of blood sugar levels through wearable sensing and timely intervention^4^. Similarly, patients with cardiovascular conditions might use wearable sensors to monitor vital signs, providing early detection of anomalies and enabling prompt medical attention^5^.

To effectively implement remote monitoring, it is essential to integrate data entries from patients and/or healthcare providers with data from wearable sensors in ambulatory settings^6^. This integration necessitates evaluating the wearable’s ability to detect specific events, such as fall detection for the elderly^7^, or identifying and validating biomarkers in real-life settings^8^. Remote monitoring has proven valuable in tracking and analyzing chronic events in certain populations, such as headache attacks of migraine patients^9–11^, seizures in epilepsy patients^12^, or depressive episodes^1^.

Given the potential of remote monitoring, there are an increasing number of studies that collect wearable data along with acute event data in ambulatory settings. However, significant challenges arise when analyzing wearable data in real-world scenarios^13,14^. These analysis challenges stem from issues related to data quality, which occur throughout participant acquisition, data collection, and retrospective analysis. They encompass problems related to participants, monitoring devices, logging applications, and technologies^15,16^. Many studies in ambulatory wearable monitoring currently either overlook or sidestep these challenges^10,11,17^. This paper aims to address this gap by offering practical and actionable countermeasures to the below-identified data quality challenges.

Specifically, we categorize the data quality challenges into two groups: those related to (i) participants and monitoring applications, and those related to (ii) wearables in real-world settings, thereby excluding other domain challenges such as technology, system architecture, and scalability^16,18^. The (i) participant and monitoring application category includes problems with data entry and quality, lack of participant compliance and motivation, unverified assumptions, and personal biases. The (ii) wearable category focuses on issues like non-wear periods, wearable artifacts, and missing wearable data.

To address (i) participant and application-related challenges, we introduce a novel participant compliance visualization technique to monitor participant motivation in near-real-time. In addition, we propose interaction-triggered questionnaires to reduce and filter data entry errors. For the (ii) wearable analysis challenges, we present an efficient and performant non-wear detection pipeline for processing wearable data at larger scales. Additionally, we propose a generic, visualization-oriented approach to validate signal processing pipelines. Lastly, we outline a bootstrapping technique to assess the variability of wearable-derived features on partially missing data segments.

To elucidate these challenges, we draw upon our first-hand experience during the mBrain study^9^. By utilizing an excerpt of this mBrain21 dataset along with the ETRI lifelog 2020 dataset, we substantiate our proposed countermeasures with reproducible implementations (https://github.com/predict-idlab/data-quality-challenges-wearables)^19^. As such, this work aims to aid future monitoring studies in bridging the gap between recognizing the occurrence of the identified challenges and the practical applicability of countermeasures.

## Related work

Over the past decade, research interest in ambulatory wearable-based monitoring studies has significantly increased, leading to multiple works indicating challenges and limitations inherent to such studies. In this section, we outline works that consider these challenges.

Schmidt et al.^20^ provided guidelines and practical implementation details from their field study to enhance the accuracy of manual data entries for ecological momentary assessments (EMA) in ambulatory wearable monitoring. They emphasized brevity in EMA duration, targeting core study goals, and daily screenings of wearable signal modalities to routinely assess data quality. This is crucial to perform timely re-instructions to participants when a decline in data quality becomes apparent. Furthermore, they suggested configuring EMA applications to match participants’ circadian rhythm and proposed incremental reward systems to sustain participant engagement. However, their work lacked practical examples of participant interventions and did not focus on providing methodologies to leverage collected ambulatory wearable data, along with EMA events, in downstream analysis.

In 2019, Schmidt et al.^13^, expanded on these guidelines by integrating wearable data processing, but did specifically put this in the context of the other challenges associated with field studies.

Balbim et al.^21^ discussed data quality challenges associated with Fitbit Physical Activity (PA) trackers, focusing on study preparation, intervention delivery, and study closeout. However, they did not address data analysis, leaving a gap in methodologies for handling data post-collection.

Cho et al.^16^ conducted a systematic review, identifying three overarching factors influencing wearable data quality: device- and technical-related, user-related, and data governance-related factors. Device- and technical-related factors include hardware issues such as sensor degradation or malfunction, software issues related to the quality of proprietary wearable algorithms, and networking issues such as data loss during transmission and recording. User-related factors involve non-wear periods and user errors stemming from wearable misplacement or poor skin contact. Lastly, data governance-related factors arise from the lack of standardization, inconsistency in algorithms across different devices, and sensor placement variations. While their review concentrated on elucidating these factors, it did not provide methodologies to address them.

Similarly, Sriram et al.^18^ identified three highly comparable factors that affect data quality; sensor or device-related, human or user-related, and system architecture factors.

Aligning with both taxonomies, this work focuses on challenges related to user-related and device-related factors, thereby omitting technical and data governance challenges.

In recent work, Böttcher et al.^22^ assessed the data quality of the wrist-worn Empatica E4 wearable, particularly in the context of epilepsy monitoring, using multiple datasets from hospitalized and ambulatory care settings. They evaluated data quality through computing signal quality scores for several physiological signal modalities of the Empatica E4 and computed a wearable-on-body score, along with a data completeness score, representing the ratio between the actual recorded volume and expected data volume. Their findings suggested superior data quality and completeness during nighttime (8 PM–8 AM). Notably, wearable streaming revealed a higher data loss compared to on-device logging.

However, their work did not provide actionable methodologies for improving data quality or conducting analysis during study collection. Additionally, while they shared analysis results publicly (GitHub, https://github.com/WEAR-ISG/WEAR-DataQuality/tree/main), documentation was minimal, and source data was not shared due to data-sharing agreements.

In conclusion, while considerable research has highlighted data quality challenges prevalent in wearable monitoring studies, there remains a significant gap in providing actionable countermeasures, tangible examples, and streamlined code for addressing post hoc analysis issues. Furthermore, few studies offer access to their data or code, complicating the assessment of their methodologies' broader applicability. By addressing these gaps, our work aims to provide practical solutions and reproducible methods to improve user and wearable-related data quality and analysis in ambulatory wearable-based monitoring studies.

## Methodology

This section outlines our approach to tackling data quality challenges. First, we introduce two distinct datasets employed to demonstrate these challenges and highlight their characteristics. Next, we define the scope of our work, distilling the specific data quality challenges we aim to address. Finally, we describe the programming environment and tools chosen to tackle these challenges.

### Datasets

We materialize data quality challenges by using examples from the mBrain21 and ETRI lifeLog 2020 datasets, whose characteristics are outlined in Table 1.

**Table 1:** Comparative overview of the characteristics of the two datasets selected for this study.

	mBrain21*	ETRI lifelog 2020
Subjects	4*	22
Country	Belgium	South Korea
Age range (median, 95% CI)	*/*	28 [21, 33]
Sex (% female)	*/*	41%
Study duration	90 days	28 days
Wearable type	Empatica E4	Empatica E4
Wearable placement	Wrist	Wrist
Recording mode	Streaming	Device
Labeling	Self-report	Self-report

*This dataset only provides an excerpt of 4 participants out of the 30 participants from the second wave of the mBrain study due to informed consent availability reasons.

The second wave of the mBrain study, i.e., mBrain21, monitored 30 patients diagnosed with chronic headache disorders over 90 days. Data from four participants who consented to public distribution were used in this work. Monitoring involved smartphone sensors (i.e., movement, application usage), the Empatica E4 wrist-worn wearable, and a dedicated logging application to record headache events, medication intake, and daily questionnaire responses^23^. mBrain21 participants were instructed to wear the Empatica device for at least 8 h per day. The Empatica E4 streamed data to the logging application, which sent it to internal servers after a two-minute buffer. This near real-time wearable data stream was utilized to construct automatic timelines of activity and stress predictions, as shown in Supplemental Fig. 1. The primary objective of the mBrain study was to analyze ambulatory wearable data in relation to headache intervals. For instance, wearable movement data was utilized to investigate changes in movement behavior during cluster headaches, as demonstrated in Vandenbussche et al.^24^*.*

The ETRI lifelog 2020 study monitored 22 participants for 28 days to acquire data-driven descriptions of human life from various perspectives^19^. Specifically, the ETRI dataset is composed of a smartphone, the Empatica E4 wearable, and Withings sleep-quality monitoring mat. Participants utilized a dedicated logging application to self-report their activity, social state (alone, with someone, with a group), semantic location (e.g., home, work), and emotional state (valence, arousal). These self-reported labels were presented to the user via a timeline. Unlike, the streaming approach in mbrain21, participants in the ETRI study were tasked with offloading the on-device logged Empatica data to a computer, which then uploaded it to Empatica’s cloud.

Both studies under consideration utilized the Empatica E4, a medically graded wristband that captures physiological and movement data. The E4 contains a three-axis accelerometer which samples at 32 Hz with a range of ± 2 g. The 64 Hz blood volume pulse (BVP) signal is constructed from a proprietary on-device algorithm that leverages the green and red exposure photoplethysmography (PPG) signals^25^. This derived BVP signal serves as input for proprietary algorithms that compute the inter-beat-interval (IBI) timings and the mean heart rate (HR). The skin surface temperature (TEMP) is acquired at 4 Hz via a thermopile sensor. Lastly, the skin conductance or electrodermal activity (EDA) is acquired at 4 Hz via two AgCl electrodes.

We deliberately selected these two datasets given our direct experience with the mBrain21 dataset and the well-documented nature and availability of the ETRI lifelog 2020 dataset. Since both datasets are recorded by different research institutes, and capture different demographic populations, we believe that they should demonstrate a certain genericity of our presented methodologies. As we were not involved with the ETRI lifelog’s data collection, we rely on examples from the mBrain study to illustrate countermeasures for participant data entry challenges.

### Selecting data quality challenges

This work focuses on challenges related to data completeness and correctness in ambulatory monitoring studies, specifically those using wrist-worn devices along with an application for ambulatory label acquisition. Our emphasis stems from first-hand experience with the mBrain project, which fits this study type. Moreover, such studies are frequently employed by smaller-scale research to assess the wearables’ potential of detecting these ambulatory labeled events of interest, such as affect, headaches, and stress^10,11,13^.

We categorize the data quality challenges into two domains, which occur within the taxonomies Cho et al.^16^ and Sriram et al.^18^: (1) participant data entry challenges, and (2) wearable analysis challenges. The first category, participant data entry challenges, encompasses participant and application-oriented challenges that impact data quality. These include participant compliance and motivation (challenge 1; C1), implicitness assumptions (C2), data entry errors (C3), and personal bias (C4). Conversely, the wearable data challenges concentrate on wearable-related analysis challenges, including wearable non-wear (C5), wearable artifacts (C6), and the analysis of “windows-of-interest” with missing or anomalous data (C7).

For each of these seven identified challenges, we provide detailed insights into their causes, impacts, and potential countermeasures. Wherever possible, we illustrate these countermeasures with concrete visualizations and implementation examples, applicable to either the data analysis side (retrospective) or the application side (prospective or reactive).

### Programming environments

Longitudinal wearable monitoring studies produce large datasets. As indicated by the Kaggle 2022 data-science survey, notebook-based environments, particularly those using IPython, are the go-to tools for data scientists^26^. Interactive notebook-based formats drive data exploration, which is crucial in every step of the data science process^27^. Consequently, this study employs IPython environments to illustrate methods for tackling data-centric challenges. The Python packages utilized in this work are listed and managed using the Poetry Python package manager^28^.

Notably, both Weed et al.^29^ and Böttcher et al.^22^ utilized MATLAB to perform their wearable analyses. However, we believe that Python’s scalability, open-source nature, larger community, and easier integration with other technologies, along with cost-effectiveness and flexibility, is a more suitable choice.

## Participant data entry challenges

In this section, human- and application-related data entry challenges of wearable monitoring studies are covered.

### Challenge 1: participant compliance

Participant motivation to wear a wearable device, interact with questionnaires, and submit events of interest is crucial for obtaining a qualitative dataset. However, as seen in numerous cases, including our own mBrain study^9^, motivation and study interaction tend to decrease over time^20,30^. This decline can be attributed to several factors, including inconvenient wearable connection processes, wearable design aesthetics, frequent or poorly timed EMA collection, and a negative user experience with the study (including adverse reactions to the wearable device).

To minimize user burden, each labor-intensive component, including wearable use and EMAs, should target the core goal of the research, with minimal overhead^20,31^. Frequent or long-term manual data entry can lead to response fatigue, reducing data quality and accuracy^32^. Maintaining participant commitment throughout the study is essential for high-quality data and labels, both in frequency and completeness^20^. Automated input processing, through digitally acquiring user data via structured forms on smartphones is a valid alternative to traditional double manual data entry^33^. Additionally, strategies such as incremental rewards^34,35^, gamification^36,37^, and periodic contact with participants^38^ can help sustain motivation. Lastly, querying participants about their experiences, feelings, and gains from participating at the study's conclusion can provide valuable insights into maintaining long-term engagement.

#### Countermeasures

From a data science perspective, several solutions can be devised to address these challenges. For instance, compliance-based visualizations have been utilized to assess participant interactions during ambulatory monitoring studies^20^.

**Figure 1:**
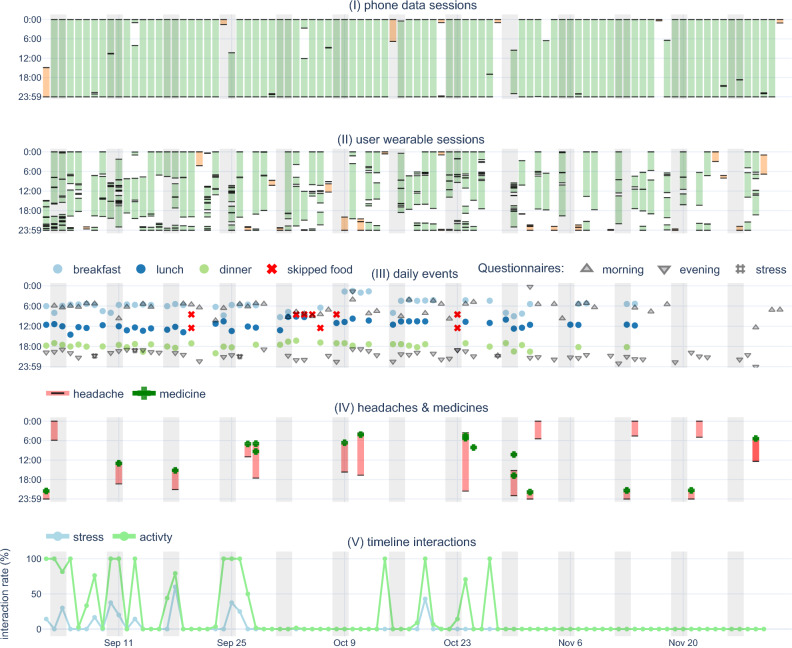
mBrain study interaction visualization of a single participant for a period of 90 days. The figure consists of several subplots with a shared x-axis, each providing different layers of information about the participant's activity and interactions. Subplots (i) and (ii) display phone and wearable data sessions over time, with each bar on the x-axis representing a unique day. For the first four plots, the y-axis indicates the time of day, revealing patterns of data fragmentation and daily volumes over extended periods. Gray-shaded areas indicate weekends. The mBrain study requires a minimum of eight hours of wearable data daily. This compliance is color-coded in the first two subplots: green represents days with more than 8 h, while orange indicates less than 8 h. The daily events subplot (iii) provides an overview of food intakes and questionnaire interactions. Subplot (iv) provides a visual record of the participant's headaches and medication intake. The final subplot (v), shows the interaction rate (%) on the y-axis, illustrating the frequency of participant interactions with stress and activity timeline events derived from the wearable data stream.

**Figure 2:**
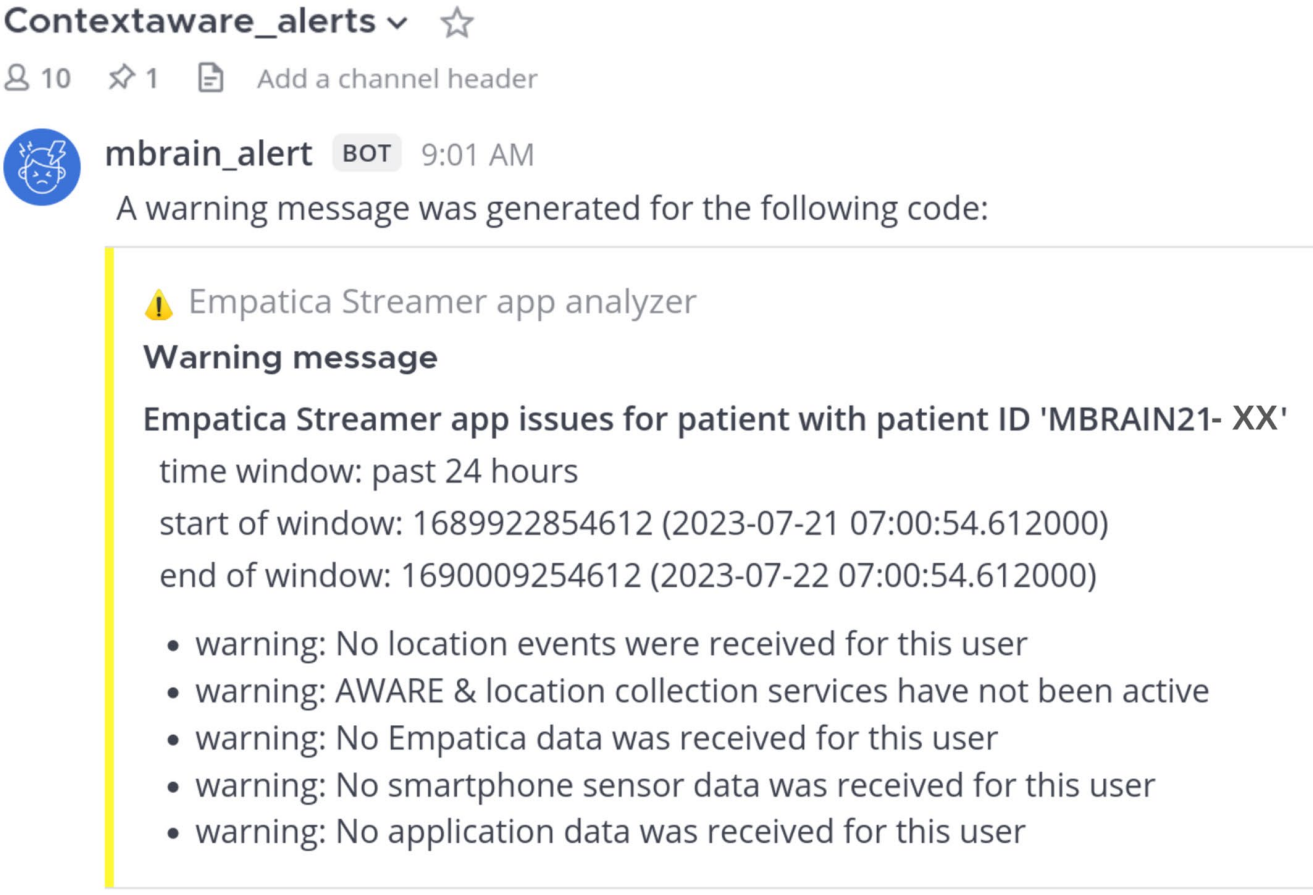
Example of an mBrain alert message, shown to the study coordinators when no wearable data is received from a participant.

During the mBrain study, we developed a methodology to evaluate participant motivation continuity by (i) automatically generating interaction rate reports from incoming data streams, and (ii) incorporating real-time notification via webhooks to alert study coordinators when participants fall below interaction thresholds. Figures 1 and 2 illustrate this methodology applied to the mBrain study. Specifically, scheduled jobs were utilized to generate these daily participant compliance reports, which, similar to visualizations from Rawassizadeh et al.^39^, displayed intervals for which there is wearable and phone data, and the evolution of answered questionnaires. This visualization provides an overview of participant compliance to study coordinators, facilitating timely re-instruction when needed. To showcase the generalizability of our proposed method, we applied this compliance visualization to the ETRI lifelog 2020 dataset (Supplemental Fig. 2).

While monitoring platforms like Empatica Care lab and REDCap^40^ offer similar features, remote health monitoring often involves various data types that cannot be stored in a single database platform (e.g., high-frequency wearable data versus survey responses). This necessitates custom solutions for a comprehensive compliance overview.

In the mBrain study, previous-day compliance data was utilized to produce daily alerts, as shown in Fig. 2, notifying study coordinators when participants logged less than 8 h of data. Based on these compliance assessments (i.e., reports and alert messages) coordinators could decide whether to contact participants to understand their reasons for reduced compliance. Within the mBrain study, the clinician-neurologist sent—whenever needed, and with caution not to overload participants as well—personalized messages to the participants via a dedicated interface. This methodology, empirically evaluated during the mBrain study, allowed physicians to track participant interactions and enhance study compliance through a built-in monitoring system with rapid notification capabilities.

### Challenge 2: implicitness assumptions

A common assumption in data collection is that the absence of a recorded event implies it did not occur^9,41^. For instance, if no medication event is logged for a day, it might be assumed that the patient did not take any medication, although the participant may have simply forgotten to log it. Therefore, it is essential to verify these implicitness assumptions through direct questioning about these (absent) self-reported events. Given the straightforwardness of this approach, it is possible that no prior studies have explicitly reported using these checks.

#### Countermeasures

**Figure 3:**
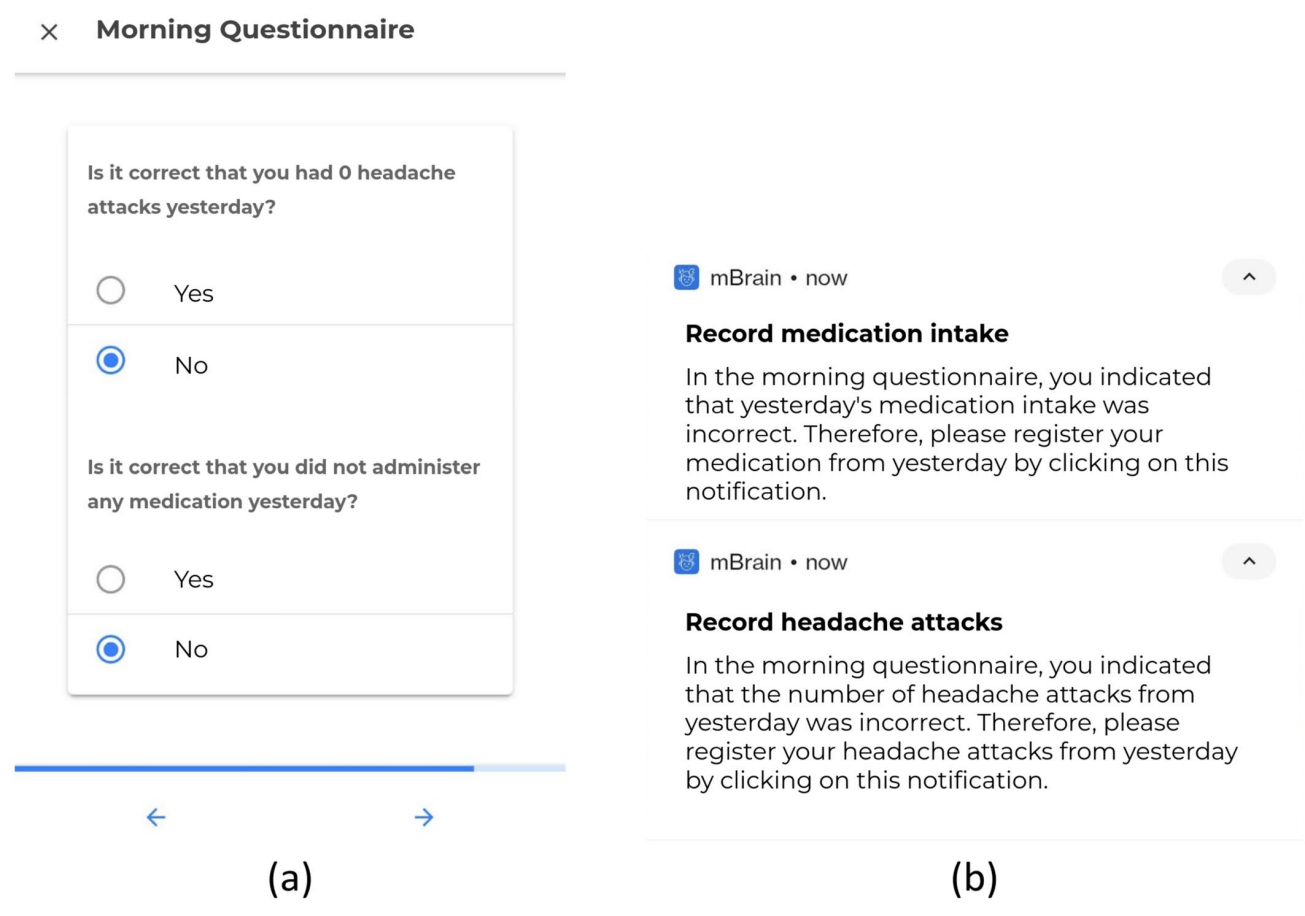
(**a**) Screenshot of questions in the mBrain study’s morning questionnaire evaluating implicitness for headache and medication events. (**b**) Notifications are activated based on responses to the implicitness questions.

In the mBrain study, morning questionnaire responses serve to validate or disprove assumptions regarding headaches and medication use from the previous day, as shown in Fig. 3a. Subplot (b) of Fig. 3 displays notifications sent to the user when their responses contradict these assumptions, such as replying “No” to one of the questions in (a). While these additional queries improve data accuracy, they also increase the burden on participants. Therefore, these checks should be confined to parameters crucial to the study's analysis. Notably, regularity in questionnaires may improve study compliance by creating a routine for the participant to interact with the study environment, and therefore possibly nudge the participant toward other components of the study^42^.

### Challenge 3: data entry errors

Data entry errors often arise from accidental user mistakes during interactions, primarily due to suboptimal design choices^43^. As such, enhancing user experience through cognitively ergonomic designs can significantly reduce these human errors^44^. A proactive strategy involves conducting a pilot phase with a small group of participants and analysts to identify and correct issues related to implicitness assumptions and data entry before full-scale monitoring^21^.

Temporal inaccuracies, a specific category of data entry errors, typically result from users’ uncertainty in allocating exact timestamps to events^20^. These inaccuracies are influenced by recall bias (misdating past events) and predictive bias (misdating future events). Notably, EMAs emerged as a strategy to evaluate immediate experiences in participants’ everyday settings, thereby minimizing recall bias^45^. Moreover, temporal accuracy can be enhanced by integrating contextual data, such as location and activities, into an automated timeline to counteract recall bias^46^.

#### Countermeasures

Within the mBrain study, we conducted two pilot phases to factor out data entry errors and assess the robustness of our infrastructure in managing higher user loads^47^. We also propose using an extensive intake procedure, where participants review all components of the logging application and the wearable device with the study coordinator. This does not only clarify the process but also benefits the participant’s motivation to perform data entries. Providing a detailed manual during the intake, in which all the intricacies of the application and study procedure are described, further supports this goal^9^. This intake procedure was successfully validated within the mBrain study.

**Figure 4:**
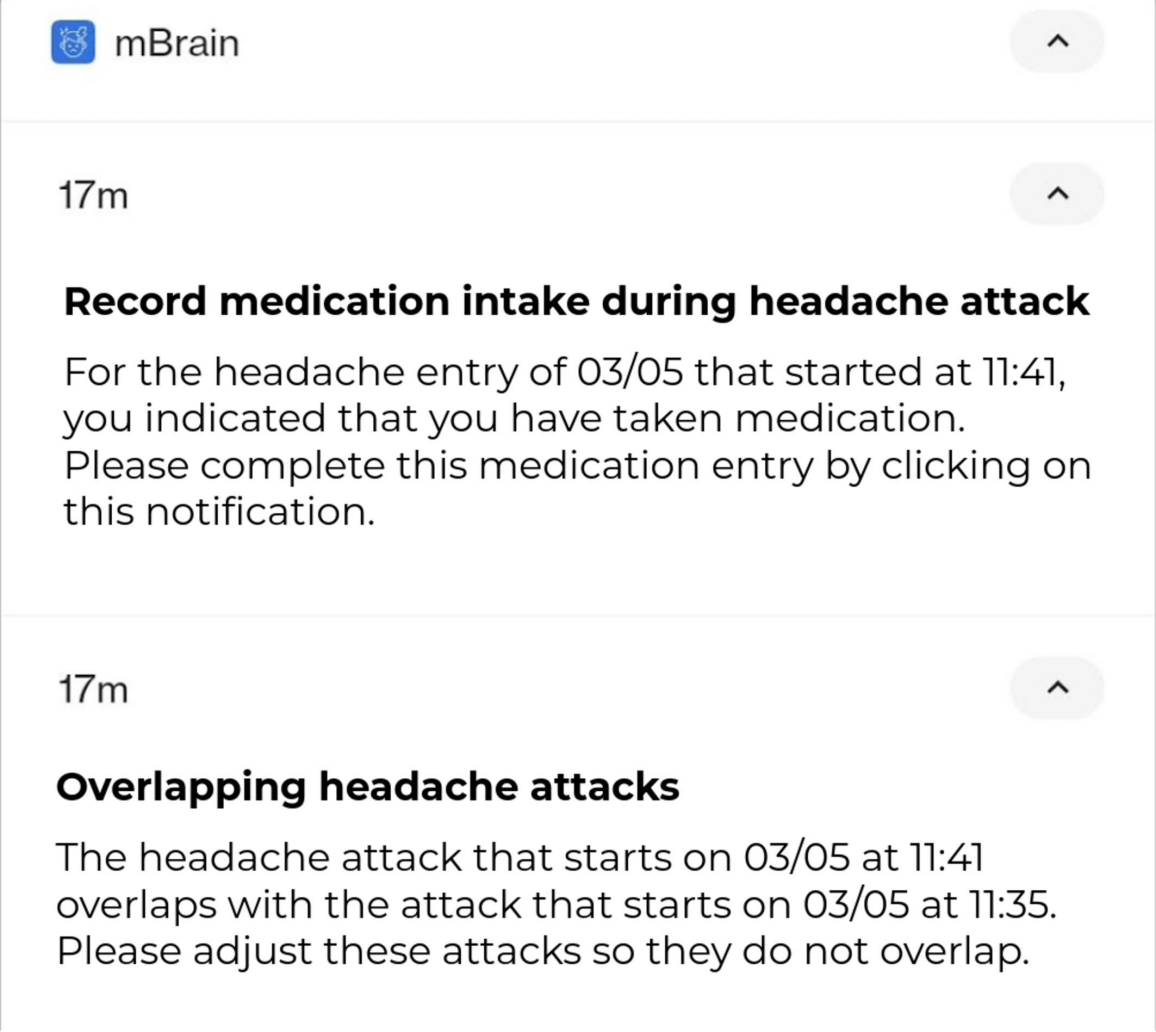
Example mBrain application notifications when conflicting data entries were made by a participant.

A reactive measure implemented based on pilot study errors includes sanity checks to reduce data entry errors. This system prevents logging multiple concurrent events of the same type and generates alerts for entries with improbable dates, such as logging a headache that occurred two weeks in the past or is set for a future date. Figure 4 showcases notifications in the mBrain study that are triggered by users’ incomplete or ambiguous data entries. Remark that these notifications may increase user burden.

To tackle temporal inaccuracies, we propose using an automated user timeline, as employed within the mBrain study, sourced from smartphone and wearable data. This timeline, as shown in Supplemental Fig. 1, improves the temporal specificity when pinpointing stress or headache occurrences. Another countermeasure is allowing participants to specify a time range instead of a single, definitive timestamp. This approach recognizes and accommodates the user's temporal uncertainty, such as by letting them denote a span for both the start and end time of a headache event. However, this flexibility might complicate the user experience and eventual analysis, so it should be carefully aligned with the study's objectives to ensure its analytical value.

### Challenge 4: personal bias

In remote health monitoring studies, where participant self-reporting plays a central role, personal bias emerges as a substantial challenge. This bias arises from the subjective nature of self-reported data and the various ways in which individual perceptions, beliefs, and motivations influence these reports^20^. Recognizing and correcting for personal biases enhances the overall validity of the study. It ensures that the conclusions drawn are genuinely reflective of the observed phenomena, not skewed by individual user tendencies or perceptions.

Addressing personal bias necessitates a multifaceted approach, including rigorous study design, participant education (to, e.g., homogenize definitions of concepts like stress), regular reminders, intuitive technology interfaces, and integrating objective monitoring tools. Researchers should always consider personal biases when interpreting subjectively labeled data.

Modeling participants as latent factors during analysis, by for instance modeling the participant as a random effect with a Linear Mixed Model, is a recommended practice^48^. Including the participant as a random effect allows for the modeling of the variability between participants and helps in accounting for the within-subject correlation due to repeated measurements on the same participant. This way, any inherent individual bias or subject-specific characteristics (like baseline levels) that might affect the outcome variable can be taken into consideration during analysis.

Beyond self-reporting, personal bias can also manifest in device wear behavior. For instance, during the mBrain study, we noted that participants wore the wearable devices less frequently during headache episodes (Fig. 8). Some participants might also avoid wearing devices during more intensive activities, potentially skewing the findings. A general countermeasure to this challenge is to instruct participants that the monitoring study aims to observe each aspect of their daily life and that they therefore must keep wearing the wearable whenever possible. Moreover, when such behavior is observed through compliance reports, participants can also be contacted during the study period (see C1).

Finally, the Hawthorne effect, i.e., the modification of participant behavior in response to being observed, can also affect data representativity^49^. It has been demonstrated that the Hawthorne effect appears to last for a limited time^50^. Hence, monitoring studies that have an adequate duration (i.e., up to 3–6 months) can mitigate this issue.

#### Countermeasures

**Figure 5:**
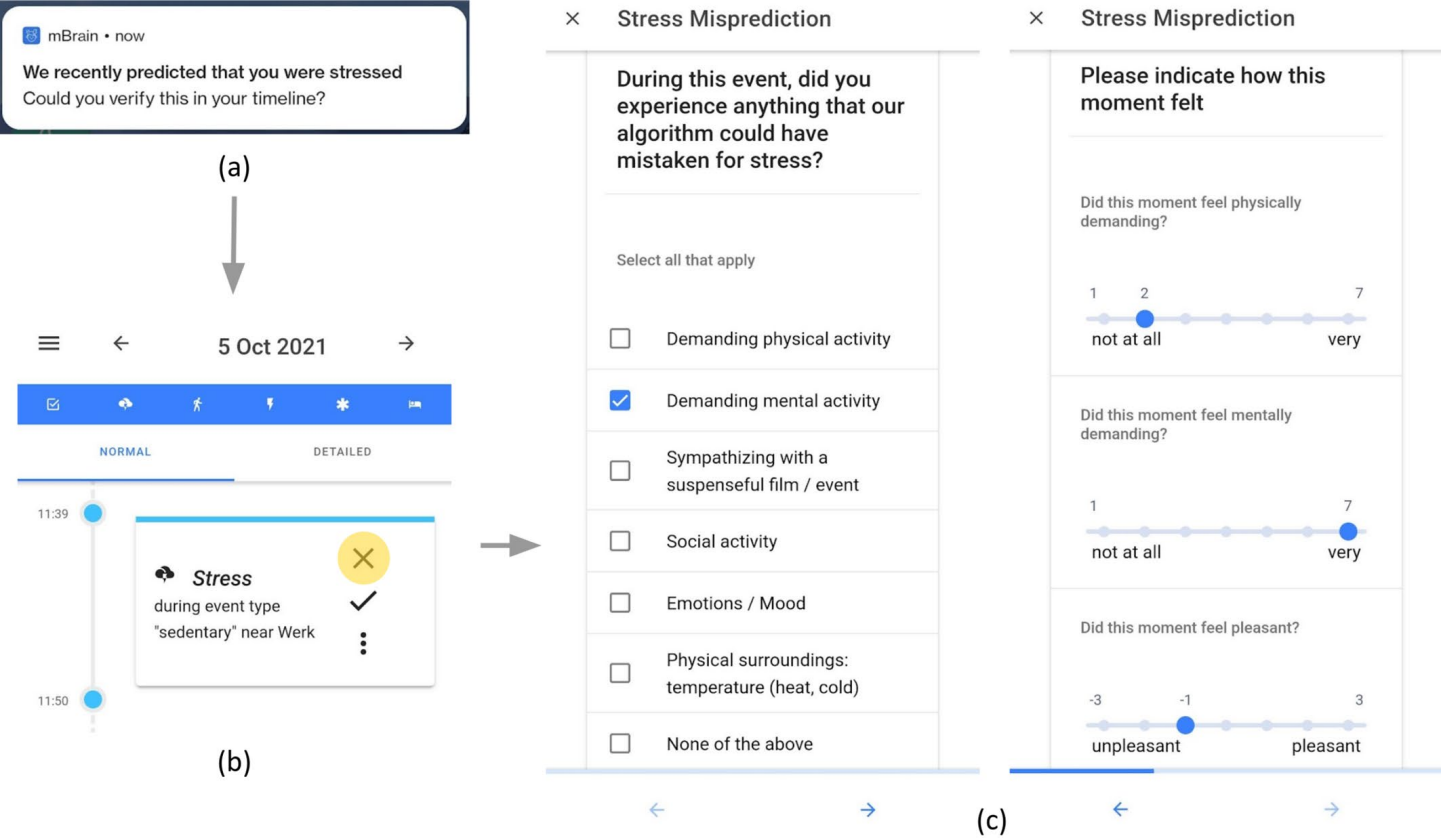
mBrain stress event interaction and its corresponding misprediction questionnaire. *Note:* When a stress-system-activation (e.g., a sudden non-activity-induced increase in skin conductance responses) is detected in the streamed wearable data, a notification is sent to the user as shown in (**a**). This notification aids in reducing the interaction latency of the participant. When clicking on this notification, the participant is guided toward the mBrain timeline in which the recent stress event is shown, as depicted by (**b**). The yellow circle indicates that the participant re-labeled the stress-event period to be non-stressful. This, in turn, prompts the participant whether they have time to fill in a questionnaire that gauges for more contextual information about this event. This questionnaire is portrayed in (**c**) and indicates that the user was performing a demanding mental activity that was not perceived as really pleasant, possibly explaining the stress response.

We propose using interaction-triggered questionnaires to gauge causes and contextual information related to the accuracy of highly personal events such as stress. Figure 5c depicts such a questionnaire from the mBrain study, demonstrating how—although the participant disproved the stress prediction—the event was perceived as mentally demanding and slightly unpleasant, which might be indicated as stress by other users.

**Figure 6:**
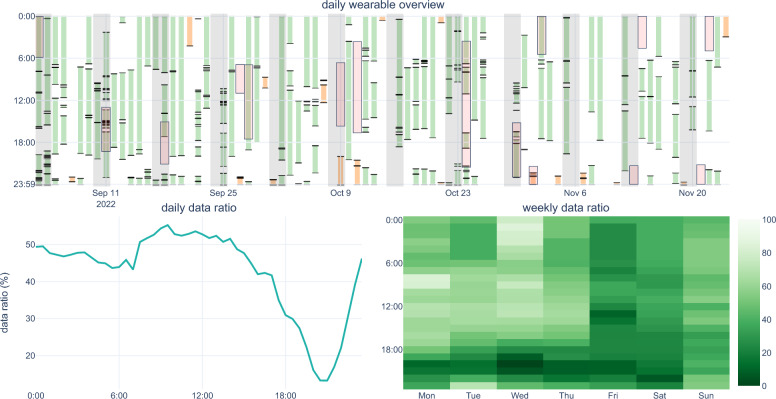
mBrain study wearable wear behavior overview of a single participant. The upper subplot illustrates the available wearable sessions, using similar bar intervals as Fig. 2, providing an overview of wearable usage. In this subplot, weekends are marked in gray, and headache intervals in red. This participant has an average wearable data ratio of 44%, whereas the available data ratio during headaches is 39%. The lower left subplot depicts the average data ratio for the time of day throughout the study period. This subgraph reveals a notable decline in wearable use between 17 h 30 and 22 h 30. Conversely, the lower right subplot utilizes a heatmap to display the average data ratio against the time of day, distributed over each day of the week, highlighting discernible patterns in wear frequency. This heatmap elucidates that this specific participant has a tendency for reduced wearable use on Fridays and Saturdays, while Wednesdays exhibit the most wearable use. Remark how the reduced wearable usage during the evening period, shown by the through in the lower left subplot, is also discernable in this heatmap visualization.

Moreover, participant interaction visualizations, as illustrated by Fig. 6 for the mBrain study, allow coordinators to observe whether trends in non-wear or non-activity (e.g., no wearable data during evening periods). Insights from these visualizations can be utilized to send custom messages to participants to gauge their behavior. By assessing this personal bias, researchers can better understand how individual differences in lifestyle, behavior, and interaction with the device might influence the data collected. This leads to a more accurate interpretation of the health metrics derived from wearable devices.

### Summary

To conclude this participant data entry section, Table 2 summarizes the four identified participant- and data entry-related data quality challenges and their corresponding countermeasures**.**

**Table 2:** Summary of the participant data entry challenges and their countermeasures.

Challenge	Countermeasures/actions
Participant compliance	
High user burden & response fatigue	*App interactions*: Minimize overhead; each component should target the core goal *Wearable*: Strive for a convenient wearable experience (e.g., connection process, battery life, …)
Decline in motivation	*(reactive)* Monitor participant compliance (+ reinstruct when needed) *(proactive)* Periodic contact with a study coordinator *(proactive)* Incremental reward systems & gamification *(retrospective)* Query experiences during takeout to pinpoint motivation hurdles
Implicitness assumptions	
Event absence assumption	(*proactive*) Utilize daily questionnaires to validate these assumptions (should always relate to core goal)
Data entry errors	
Application entry errors	*(proactive)* Pilot phase to factor out errors *(reactive)* Sanity checks & notifications
Temporal inaccuracies	*(proactive)* Providing contextual data reduces recall bias *(proactive)* Gauge for temporal certainty
Personal bias	
Labeling bias	*(reactive)* Gauge for contextual information/reasoning *(retrospective)* Include participant effect during analysis
Wear behavior	*(proactive)* Instruct participants to wear the device all the time *(reactive)* Monitor wear behavior and interfere when needed
Hawthorne effect	Monitor for a sufficient duration (e.g., 3–6 months)

## Wearable analysis challenges

This section addresses challenges related to the quality of ambulatory wearable data in the context of performing data analysis. The objective of such data analysis is primarily to examine “windows of interest”, which can include event-related time spans (such as headache periods) or specific intervals of the day (like nighttime). We identify three key challenges: (1) data streaming when the wearable is not worn (C5), (2) artifacts introduced by the wearable device (C6), and (3) strategies for analyzing wearable data that includes missing or spurious data (C7), arising from scenarios like non-wear and device-generated artifacts.

### Challenge 5: wearable not on body

Non-wear periods, where the wearable records data despite not being worn, have long been acknowledged as a crucial challenge in actigraphy research^51,52^. To address this, a variety of approaches have been established, which utilize wearable movement (ACC) signals for detecting non-wear. This detection is often performed as a preprocessing step, filtering the data before further analyses. Ahmadi et al.^53^ evaluated five non-wear detection algorithms using only wrist-worn accelerometer data, finding that standard deviation-based algorithms effectively detect non-wear periods that last at least 30 min. However, this granularity may be insufficient when aiming to analyze specific time-located events.

Recently, there has been an increase in the development of non-wear detection algorithms that incorporate physiological parameters, such as skin temperature and skin conductance, in addition to wearable movement. Vert et al.^54^ utilized the GENEActiv wrist-worn wearable, which includes a near-body temperature sensor along with a light sensor. By utilizing the rate-of-change of the temperature signal, their algorithm is able to detect non-wear periods for intervals as short as 5 min. Remark that this high temporal specificity is unattainable when exclusively using movement signals. Vert et al. also emphasized the importance of detecting such shorter non-wear periods in free-living scenarios, which often include short removals, e.g., when showering or washing hands. Similarly, Pagnamenta et al.^55^ integrated temperature data into their non-wear detection algorithms for the Axivity AX3 wrist-worn wearable. They used a relative temperature threshold of 3 °C to identify non-wear periods for 5-min windows, achieving high sensitivity and specificity compared to algorithms relying solely on accelerometer data. Lastly, Böttcher et al.^22^ developed an on-body score for the Empatica E4, combining the skin conductance, skin temperature, and movement signals. This binary on-body score is computed for 1-min intervals and assesses data quality in retrospective datasets. However, these approaches often require hyperparameter configuration specific to the wearable and climate, limiting their generalizability. Additionally, only limited efforts have been made towards optimizing these algorithms.

#### Countermeasures

Given that both datasets under consideration in this work utilize the Empatica E4, and previous studies indicated enhanced accuracy when incorporating physiological signal modalities, we refine Böttcher’s algorithm to be more efficient and sensitive*.* Table 3 compares parameter values for both algorithm versions.

**Table 3:** Algorithmic and parameter-based comparison of two non-wear detection algorithms.

	Böttcher et al	Refined (ours)
Movement SQI	ACC-SD sum (SD window = 10 s) ≥ 0.2 g	ACC_x-SD (SD window = 1 s) ≥ 0.1 g
Skin temperature SQI	25 °C ≤ *valid* ≤ 40 °C	≥ 32 °C
Skin conductance SQI	> = 0.05 μS	≥ 0.03 μS
SQI processing	1-min mean per SQI ≥ 1% on-body → *valid*	Reindexing *(i.e., ensuring a shared index)*
SQI aggregation	OR-aggregation	OR-aggregation smoothing
Inference	38 ms per hour(*)	6 ms per hour(*)
Granularity	1 min	0.25 s

*Both inference timings were computed on the same hardware. A reference notebook with both implementations and timing details can be found https://github.com/predict-idlab/data-quality-challenges-wearables/blob/main/notebooks/mBrain/C5_wearable_off_wrist.ipynb.

Specifically, we simplified the movement standard deviation computation by considering only the x-axis of the ACC signal, as opposed to calculating the standard deviation for all three axes followed by a summation. This simplification improves the efficiency, as ACC-based operations proved to be the bottleneck of Böttcher’s algorithm, given its high sample rate (32 Hz). Using a smaller window size for the sliding window SD computation (1 s instead of 10 s) further enhanced efficiency. Empirical validation indicated a high correlation between the simplified and the original ACC-SD signal. Next on, in alignment with the work of Böttcher, the ACC-SD signal is transformed into a binary Signal Quality Index (SQI) by using a threshold value. Both versions employ thresholding for skin conductance and temperature, resulting in signal-specific SQIs, with different empirically determined threshold values.

After this step, our approach deviates more from Böttcher’s algorithm. In particular, Böttcher aggregates each of the three SQIs to a binary value per 1-min window by determining whether more than 1% of that SQI is considered on-body. Subsequently, these three 1-min SQIs are combined into a single binary SQI that becomes on-body if at least one of the signals is on-body (OR-operation).

**Figure 7:**
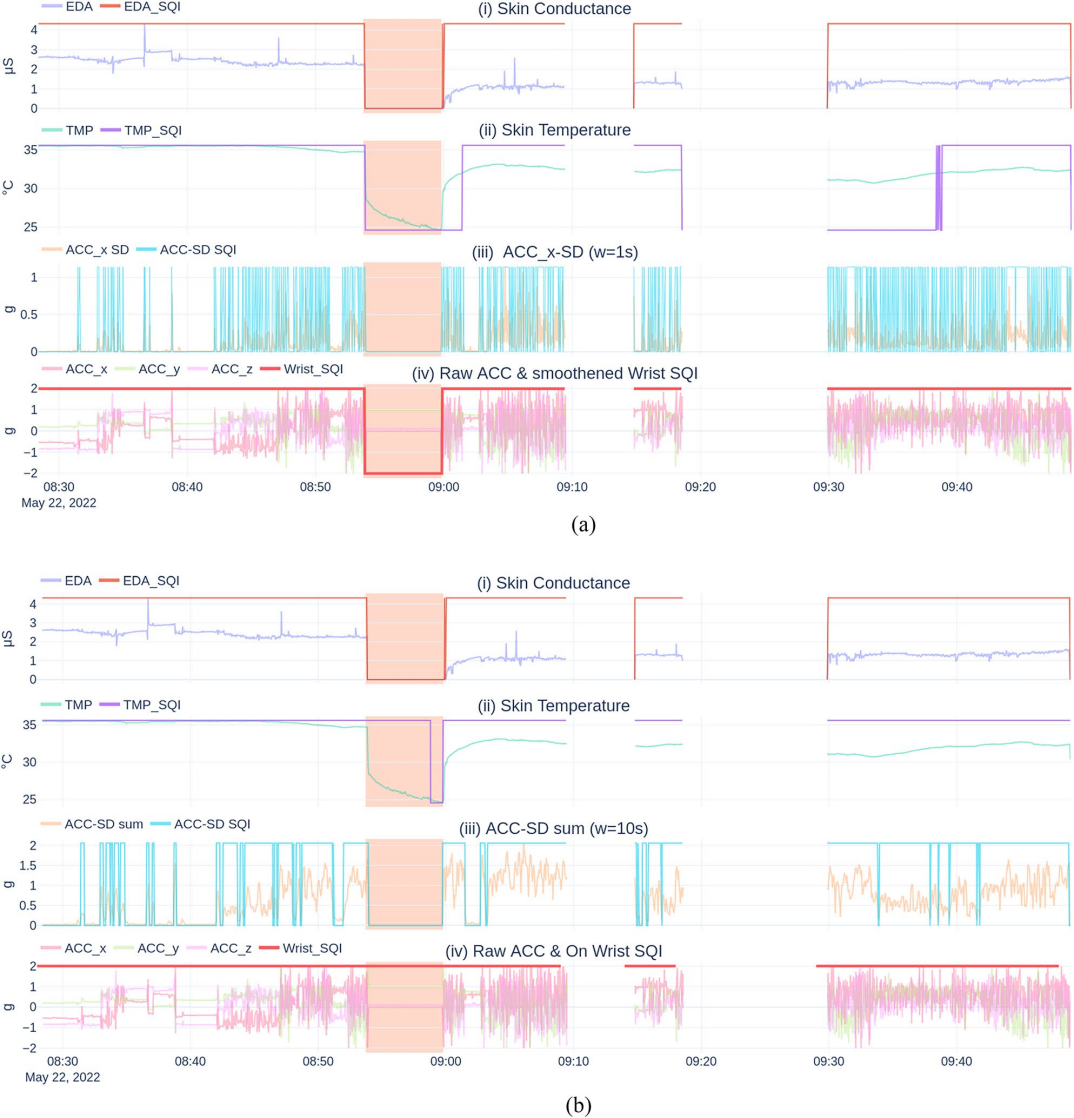
Visual comparison of Böttcher’s and our refined non-wear detection algorithm on the same excerpt. (**a**) Our refined non-wear algorithm. (**b**) Non-wear algorithm of Böttcher et al. The red-shaded area in each subplot of both (**a**) and (b) represents a labeled non-wear interval. Subplot (i) and (ii) in figure (**a**) and (**b**) depict the signal-specific SQIs for the skin conductance and temperature, while subplot (iii) represents the standard deviation of the ACC and the corresponding ACC-SD SQI. Subplot (iv) shows the three-axis accelerometer data alongside the resulting “Wrist_SQI”. A low Wrist_SQI value between 08 h 55 and 09 h 00 in subfigure (**a**) denotes non-wear. Examining this time interval in subplots (i) and (ii) of (**a**), a notable decline in skin conductance and temperature is observed, leading to low SQI values. Minimal movement within this interval also reflects a low SQI value in subplot (iii). Conversely, in subfigure (**b**), this non-wear bout remains undetected, primarily due to the valid temperature SQI range (i.e. between 25 and 40 °C). This lower bound may be set too low, as only the last part of the skin temperature segment during this non-wear period results in a low SQI.

Conversely, our approach first ensures that the three SQIs are aligned by reindexing them to the timestamps of the skin conductance SQI signal (4 Hz). Subsequently, in line with Böttcher, the three SQIs are combined via an OR-operation. This combined SQI signal is then smoothed using a 1-min window, factoring out brief instances of wear and non-wear misdetections. This results in our final “Wrist_SQI”, illustrated in Fig. 7a.

Figure 9 compares Böttcher’s and our non-wear detection algorithms, highlighting Böttcher’s lower skin temperature sensitivity. Further implementation details of both algorithms can be found on GitHub (https://github.com/predict-idlab/data-quality-challenges-wearables/blob/main/notebooks/mBrain/C5_wearable_off_wrist.ipynb). When tested on a consumer-grade desktop (AMD Ryzen 2600x), our refined non-wear detection pipeline demonstrated an inference time of 6 ms per hour of E4 wearable data, a substantial improvement from Böttcher’s 38 ms (Table 3). This efficiency is crucial for longitudinal studies analyzing months of data for numerous participants or when using constrained devices. Additionally, our pipeline provides predictions with 0.25-s granularity, in contrast to the 1-min coarseness of Böttcher’s algorithm. Supplemental S3 assesses the non-wear detection accuracy of both algorithms using a labeled subset from the mBrain dataset.

### Challenge 6: wearable artifacts

Wearable artifacts, which cause spurious signal values, primarily result from either external factors (e.g., environmental noise, humidity) or sensor degradation. Unlike the controlled conditions within laboratory studies, ambulatory research is subject to varying external conditions, thereby requiring methodologies to identify or mitigate impacted modalities to account for such artifacts. Among these, motion-induced artifacts are the most prevalent and have a notable impact on the wearable's physiological modalities, including photoplethysmography and skin conductance signals^13^. Improper use of wearable devices, often due to not adhering to the recommended wearing guidelines, can also lead to artifacts, as observed by Stuyck et al.^56^. Additionally, sensor degradation, such as the polarization of skin conductance electrodes, is another major source of artifact generation^1,57^.

Since motion artifacts are a primary cause of signal corruption in wearable devices, numerous studies have turned to nighttime data as a means to mitigate this. Böttcher et al.^22^ demonstrated that data collected between 8 PM and 8 AM exhibited substantially higher quality than daytime data. Siirtola et al.^10^ conducted a wearable monitoring study, using the Empatica E4, on migraine patients to predict the likelihood of a migraine attack within the next day. They explicitly relied on nighttime data to compute reliable features. Uchida et al.^58^ found that the median skin temperature acquired during the night via wrist-worn devices can indicate the fertility phase in women, demonstrating the reliability of nighttime data.

**Figure 8:**
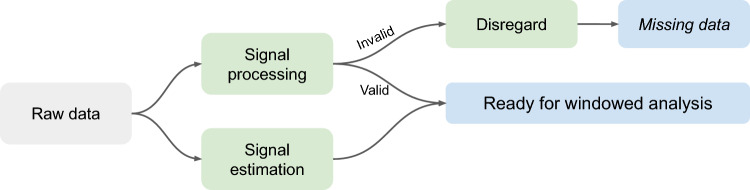
Flowchart for handling artifacts in raw ambulatory (daytime) wearable data.

However, the efficacy of relying solely on nighttime data may vary depending on the study’s specific objective. For objectives such as just-in-time interventions or analyzing physiological responses during daytime events (e.g., stress episodes or headaches), daytime data is crucial. In these cases, techniques like signal processing or signal estimation, depicted in Fig. 8, can improve data analysis reliability.

Signal estimation leverages data-driven algorithms to enhance data quality by predicting or extracting a signal from noisy data. For instance, Reiss et al.^59^ used spectral representations of the Empatica E4’s PPG signal to estimate the average instantaneous heart rate over 8-s intervals. However, a limitation of signal estimation methodologies is that they often replace the original signal without indicating the reliability of their estimations and typically require a reference ground signal truth which is leveraged by the data-driven technique^60^.

In contrast, signal processing refines raw signals into more usable data for further analysis. Unlike signal estimation, signal processing often includes validity scores and is generally more interpretable. Consequently, our research emphasizes the visual application and analysis of signal processing techniques.

#### Countermeasures

**Figure 9:**
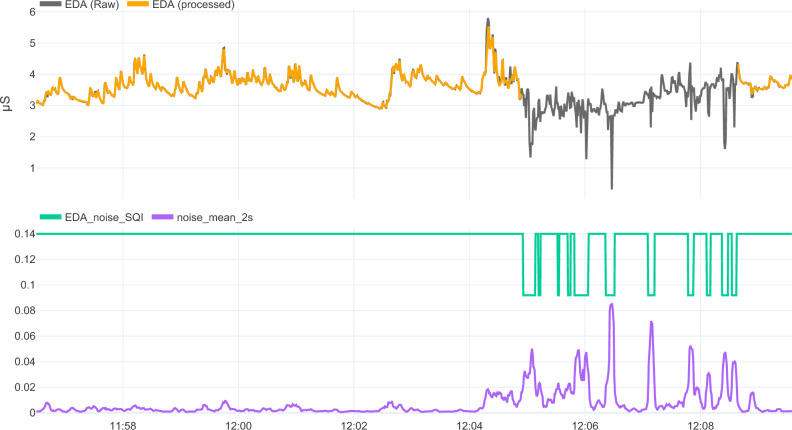
Skin conductance signal processing to discern valid and invalid regions and the resulting processed signal. The figure consists of two vertically stacked subplots that share the same x-axis. The upper subplot displays the raw EDA signal depicted by the gray line, with valid and invalid SQI regions distinguished by green and red backgrounds, respectively. The processed EDA data is illustrated by an orange line. Remark that there is no one-on-one relationship between the processed EDA data and the valid regions. This is because the duration and frequency of these invalid regions affect the eventual retention of the raw EDA signal. Specifically, brief and infrequent invalid segments, like those until 12h05, can be effectively imputed using interpolation, resulting in no data exclusion in the processed EDA signal. Conversely, as the frequency and/or duration of invalid segments increases, evidenced between 12 h 05 and 12 h 06, successful interpolation is compromised, resulting in disregarding these invalid regions. Moreover, processed EDA segments, but shorter than 60 s (e.g., valid segments between 12 h 06 and 12 h 08), are excluded given their limited analytical utility. The lower subplot elucidates the components of the skin conductance SQI. In alignment with the non-wear detection pipeline, multiple sub-SQIs are utilized. The noise amplitude of the EDA, averaged over a two-second window, is delineated by a purple line. This signal is thresholded to determine the noise sub-SQI, marked by the green line.

In alignment with our non-wear detection, signal processing solutions often utilize SQIs to differentiate valid from invalid segments. Visualizations are instrumental in shaping and evaluating these pipelines. As such, we introduce a generic visualization approach that we frequently employ through a skin conductance processing use case, shown in Fig. 9.

Essentially, our approach utilizes multiple vertically stacked subplots, all sharing a common x-axis that denotes time. The uppermost subplot displays both the raw and processed signals, enabling a direct visual comparison. In this subplot, background shading accentuates the SQI outcome, simplifying the distinction between valid and invalid segments and their impact on the processed signal. Subsequent subplots provide insights into the components used in the processing pipeline, illuminating the composition of the final SQI seen in the upper subplot.

Notably, the visualization displayed in Fig. 9 is realized by employing our widely adopted open-source Python tools. The processing pipeline is constructed using *tsflex,* an efficient toolkit that offers functionality to wrap and serialize data processing functions for time series data, facilitating convenience and easy deployment^61^. The visualization is rendered with Plotly-Resampler, a highly scalable time series visualization tool, which facilitates back-testing on large amounts of data^62^. It is this interplay between efficient signal processing and scalable interactive visualization that drives thorough analysis and broad exploration of large data volumes^63^.

### Challenge 7: missing wearable data

In ambulatory studies, encountering missing segments of wearable data is inevitable. Missing data can stem from non-random processes where the likelihood of missing data depends on other unobserved symptoms, such as the presence of severe symptoms or certain periods of the day (e.g., morning showers)^64^. Another non-random factor that may contribute to missing data is the type of activity that is performed, as certain activities may contribute towards increased artifacts, thereby introducing more missing data during signal processing (see Fig. 8). Next to these two non-random sources of missing data, device particularities can contribute to random missing data, where the missing data likelihood is unrelated to any, possibly latent, factor. For instance, during the mBrain study, the absence of an automatic reconnection functionality for the E4 device led to data loss whenever Bluetooth connectivity was disrupted. Users had to manually restart and reconnect the device, resulting in reduced data volume compared to on-device logging, even further compounded by the increased battery consumption due to Bluetooth streaming^22^.

Given the inevitability and the high prevalence of missing wearable data in ambulatory studies, its impact on study results should be considered.

#### Countermeasures

**Figure 10:**
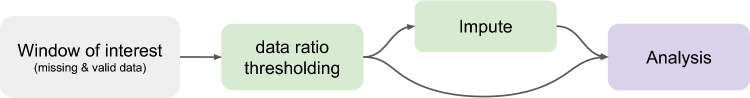
Flowchart illustrating the methodology for performing data analysis using incomplete data.

In the previous subsection, we outlined how signal processing and signal estimation methodologies can be utilized to address spurious data segments, leading to enhanced or excluded segments. The resulting processed data is then suitable for qualitative analysis. As depicted in Fig. 10, we particularly focus on “window-of-interest”-based analyses. This approach is widely employed in wearable monitoring research to partition collected data into windows for which the analysis will take place^10,11,65^. These analysis windows should be derived from the study’s research question. For example, if the study aims to understand the precursors of headache attacks, the window-of-interest could include wearable data from the day before an attack^24^.

**Figure 11:**
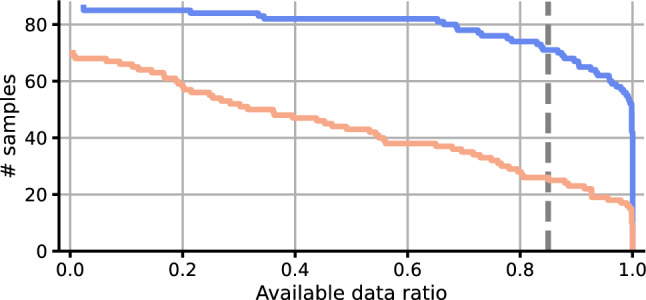
Complementary cumulative distribution plot of the window-of-interest data ratios for two participants. The y-axis represents the number of available window-of-interest samples, while the x-axis indicates the corresponding data ratio. Each curve in the plot represents the complementary cumulative distribution of a participant, providing a visual assessment of overall data availability per participant. Furthermore, when utilizing a data-ratio threshold, exemplified via the dashed vertical gray line for the data-ratio of 0.85, this visualization allows determining the remaining number of samples adhering to this threshold.

After defining these “windows-of-interest”, one can evaluate the proportion of missing and valid data within them. Figure 11 presents a complementary cumulative distribution plot of two participants, showing data availability across different data ratios. From this plot, one can easily interpret the number of samples that are available for a given data ratio per participant.

A possible countermeasure to this missing data is imputation, which replaces missing values with aggregated imputation values^66–68^. Weed et al.^29^ validated multiple imputation techniques for a 5-day window computation of actigraphy metrics using wearable movement data, finding that median same-time-of-day imputation yields the best results. A recent survey of Di et al.^64^ provides an overview of the most common imputation strategies applied to digital health time series data, also highlighting the efficacy of time-of-day-based imputations and the rise of deep learning in this domain.

**Figure 12:**
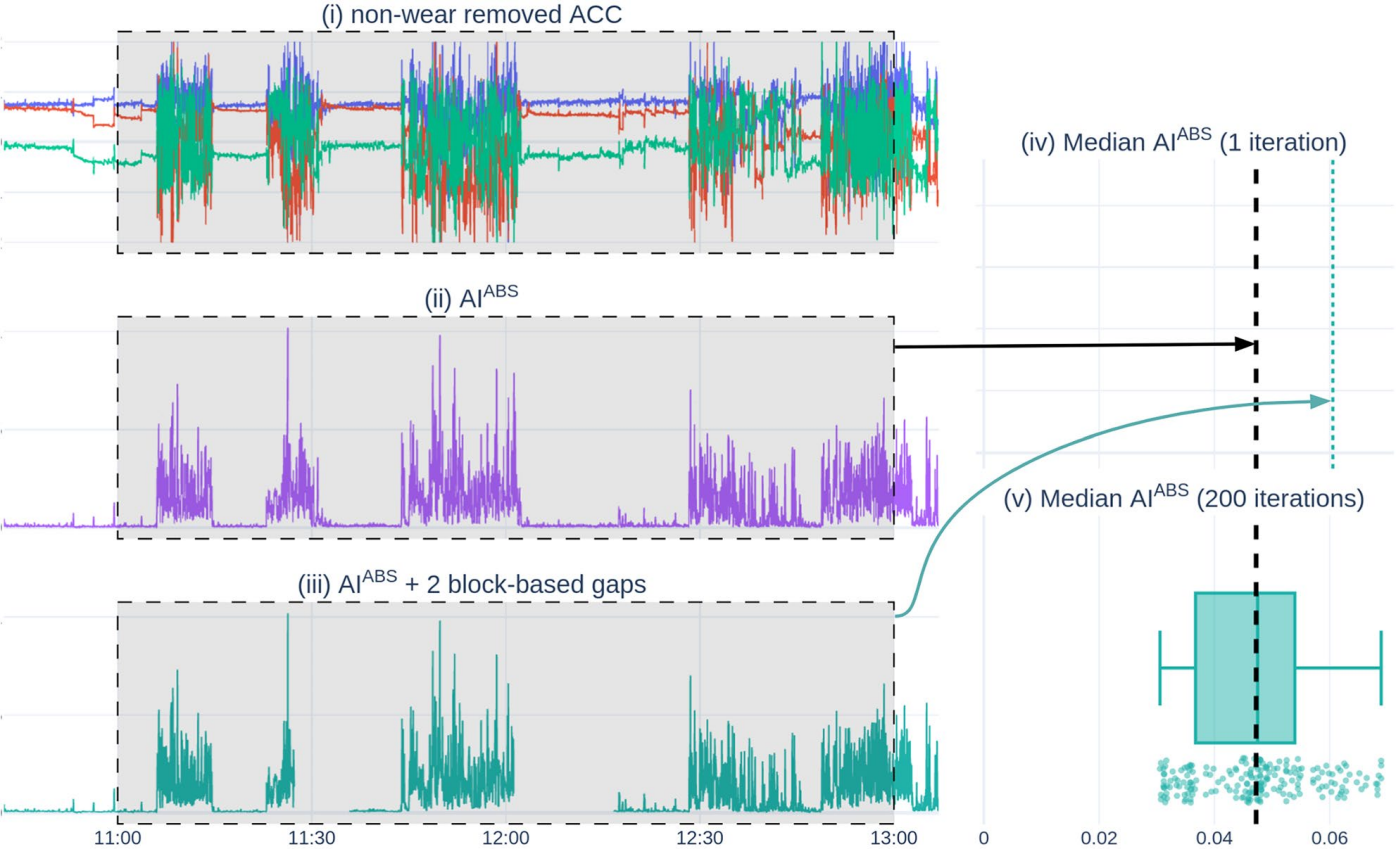
Overview of a single block-based bootstrapping iteration using the median as desired metric. The figure comprises three vertical stacked subplots on the left that share an x-axis, with the window-of-interest highlighted by a gray-shaded area. The two vertical subplots on the right side share an x-axis as well. Subplot (i) depicts an excerpt of processed wearable movement data, for which non-wear periods have been removed. Remark that no non-wear periods were detected, and no data is missing, resulting in a *complete valid segment* for our window of interest. Subplot (ii) visualizes the transformed ACC data of (i) into a second-per-second activity intensity index, AI^ABS^, in accordance with Bai et al.^71^. This AI^ABS^ signal is then utilized to compute our desired metric values, specifically, the median value of all data within our window of interest. This *reference* metric value is represented by a bold dashed black line in subplot (iv) and (v). Subsequently, gap-based bootstrapping is employed utilizing the complete movement intensity data from subplot (ii) as input. In particular, one or multiple block-based gaps are generated to create a gap-induced signal, shown in subplot (iii), maintaining a specific retention data ratio, which is in this illustration 0.6. The modified signal is then used to compute the desired metric, which is depicted by the vertical green dotted line in subplot (iv). Each bootstrap iteration results in adding another data point in subplot (v), which can then be utilized to assess the spread for a given data retention ratio. Further specifics can be found on Github (https://github.com/predict-idlab/data-quality-challenges-wearables/blob/main/notebooks/mBrain/C7_missing_data.ipynb).

To rigorously assess the impact of missing data segments on analytical results, it is generally recommended to utilize bootstrapping combined with gap simulation^69,70^. However, such analyses are often neglected, as literature tends to exclude “windows-of-interest” that contain missing data^29^. In addition, there is limited research available on data imputation for variable and short-term (i.e., sub-day) intervals, such as stress events or headache periods^29^. We are therefore among the first to introduce a detailed procedure for assessing the impact of missing wearable data on outcome metrics, as such allowing the inclusion of windows with missing data. Figure 12 illustrates this procedure using a wearable accelerometer signal.

Starting with the window-of-interest, the first step entails selecting a processed, gap-free series and then computing the analysis metrics to obtain the *reference* metric values. For a wearable movement use case, as exemplified by Fig. 12, this step is illustrated in subplots (ii) and (iv).

The second step involves gap bootstrapping. Specifically, one or multiple gaps are induced to achieve a certain data retention ratio. The gap induction method should mimic how missing data appears in other incomplete windows of interest. When dealing with wearable data, arbitrarily removing points is illogical. Instead, block-based gap induction methods which represent non-wear bouts are recommended^69^. We provide a comprehensive comparison of gap induction methodologies applied to wearable data bootstrapping in Supplemental S4.

To facilitate statistical analysis, multiple repeats of the second step are conducted on each chosen, processed, and complete reference series. This yields a set of metric values under varying simulated gap conditions, allowing observing the distribution and spread relative to the reference (gap-free) metric value, as shown in subplot (v) of Fig. 12.

**Figure 13:**
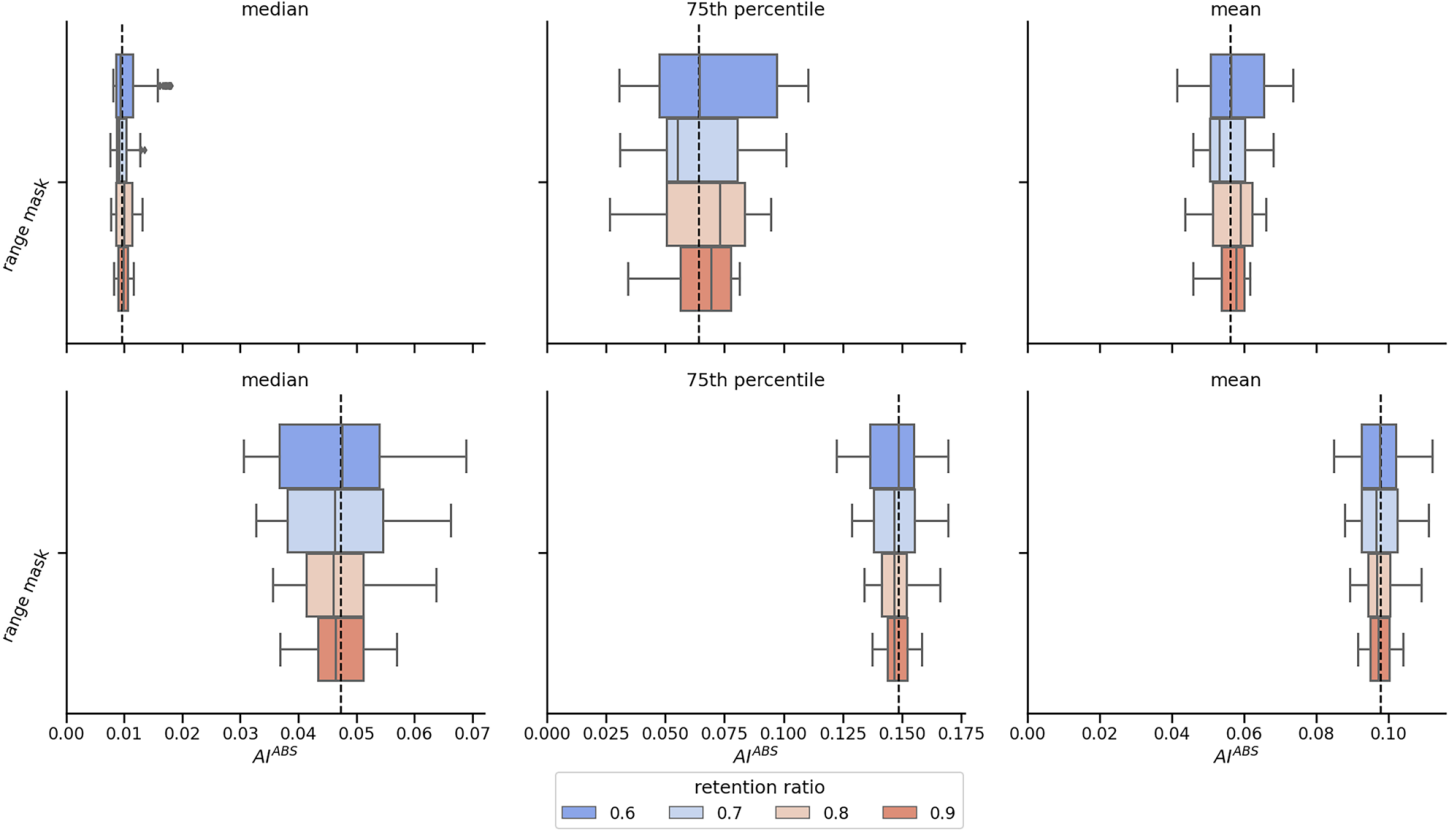
Spread analysis of block-based gap bootstrapping for various data ratios and metrics. Each row in the figure represents a distinct reference series, signifying a window of interest from a unique moment. Different columns correspond to varying metrics, with the vertical dashed black line illustrating the metric value of the gap-free reference series. In creating this specific visualization, the accelerometer data from the Empatica E4 was transformed into a second-by-second activity index, AI^ABS^, as per the methodology detailed by Bai et al.^71^ and illustrated in Fig. 12. The considered metrics are the 50th percentile, 75th percentile, and mean values calculated from the AI^ABS^ data of the selected time window.

When gaps of varying data retention ratios are simulated, we can explore the impact of the data retention ratio on metric variability. Swarm plots or box plots can visualize this by showing distributions for each combination of metric, data retention ratio, and reference series, as depicted in Fig. 13. Used alongside the cumulative data-ratio plot in Fig. 11, this facilitates data-driven decisions regarding the data retention ratio threshold for windows-of-interest.

Remark that the proposed bootstrapping analysis has certain limitations. A significant drawback is that the gap induction procedure does not account for potential biases related to the specific times when participants are not wearing the watch, as outlined in *Challenge 4 (bias)*. Conversely, solely including complete windows of interest, which is common practice in literature, may also introduce certain biases in the downstream analysis.

Furthermore, these analyses are more representative when all windows-of-interest occur at fixed time spans (e.g., the wakeful period from 10 AM to 8 PM on the day prior to a headache event), instead of varying day-time ranges (e.g., three hours before a stress event). This is because the time of day influences the occurrence and nature of gaps in the data, as indicated by Weed et al.^29^.

As mentioned earlier, imputation is a viable option for dealing with partially missing data. The impact of imputation methods can also be analyzed using our proposed bootstrapping spread analysis methodology.

For temporal cyclical data, such as circadian dependent data, cosinor-based rhythmometry may prove useful as it is a gap-robust methodology that can deal with imbalance^72,73^. Lastly, It is also advisable to consult literature to cross-reference the data ratios employed in prior research, if available.

### Summary

To conclude the wearable data quality section, we summarize the presented three challenges and their countermeasures in Table 4.

**Table 4:** Summary of wearable data quality challenges and their countermeasures.

Challenge	Countermeasures/actions
Wearable non-wear	Perform non-wear detection as a preprocessing (data filtering) step
Wearable artifacts	steer clear off: Utilize nighttime data (overall higher data quality) *Signal processing*: discern validity of signals (& enhance) *Signal estimation*: estimate target signal *Visualizations of signal processing and estimation steps are crucial for quality assessment*
Missing and spurious data	Visualize available (processed) data retention ratios for participants *Computing metrics with gaps or imputation*: Bootstrapping techniques aid in assessing the spread of your outcome metric for a given data ratio *note*: Bootstrapping can also aid in analyzing the spread of imputation techniques

## Limitations

In this work, we addressed seven data quality challenges in ambulatory wearable monitoring studies, focusing on issues related to participants, monitoring applications, and wearable devices. While we touched upon participant-related aspects, including user burden and application experience, we did not extensively explore psychological dimensions. For example, intrinsic motivation, which significantly influences study engagement, was not covered^74^. Therefore, psychological aspects should also be considered in longitudinal studies.

We recognize that our study did not quantitatively measure the impact of several countermeasures beyond non-wear detection. While certain mBrain study design choices, such as the specific validation questions used for data completion, were made based on insights available at the time, their effects on data quality and availability were not systematically assessed through methods like A/B testing. This limitation restricts our ability to generalize the benefits of these optimizations to other settings, as they may be influenced by social, technical, or other biases specific to the mBrain study.

Regarding wearable-related challenges, we focused on introducing innovative methodologies targeting wearable data quality, especially for daytime-based analyses. However, our wearable-related countermeasures are not devoid of limitations. Both the ETRI and mBrain datasets rely on the Empatica E4 wearable device, constraining our analytical examples to a single device that has been discontinued. While we believe that most of our countermeasures are wearable agnostic, device-specific characteristics might affect data quality and subsequent analyses. Via this notebook (https://github.com/predict-idlab/data-quality-challenges-wearables/blob/main/notebooks/EmbracePlus.ipynb), we aim to showcase a certain generality of our non-wear and signal processing pipelines towards the Empatica EmbracePlus device, which is the successor of the E4. Future research should extend our methodologies to a diverse range of wearable devices, including smartphones and chest-strap wearables.

Another limitation is that we did not explore wearable synchronization extensively since only a single wearable was utilized in both studies. In the mBrain study, the Empatica E4 device was connected to the phone, whose timestamp was used to synchronize the Empatica, thus mitigating the smartphone and wearable synchronization challenge. However, this challenge is addressed in literature, such as the work of Wolling et al.^75^, which provides a methodology for synchronizing multiple wearable devices that share a highly correlated signal, such as heart rate.

We also refrained from discussing the measurement sensitivity of certain wearable device types. For instance, if the objective of an ambulatory wearable study is to investigate activity patterns in participants, wrist-worn devices tend to be less accurate than chest or hip-based wearables in capturing Activity Energy Expenditure (AEE)^76^. Milstein et al.^77^ specifically evaluated the reliability of the Empatica E4’s skin conductance signal using the MindWare Mobile Impedance Cardiograph device to acquire palm skin conductance data as reference. Their results concluded that the E4 was not able to produce reliable EDA data, which may be attributed to lower sweat gland density on the wrist compared to the hand palm^78^. Therefore, it is paramount during study design to first consult literature regarding the measurement sensitivity and limitations of your device at hand.

Lastly, our work focused on enhancing the data quality during collection and processing, without explicitly addressing the impact of these steps on model training and decision-making.

In summary, while our research offers valuable insights and methodologies for improving wearable data quality, it is crucial to consider its limitations and the need for future research to validate and extend its applicability and robustness.

## Conclusion

Recent advancements in wearable sensing, particularly wrist-worn devices, offer promising solutions for longitudinal follow-up of chronic patients by shifting from intermittent, subjective self-reporting to objective, continuous monitoring. However, integrating and analyzing wearable data with health-related records presents unique challenges. We distinguished two main categories of data-quality challenges; (i) participant- and data-entry-related challenges, and (ii) wearable-related analysis challenges.

For each identified challenge, we provided insights into the causes, effects, and countermeasures. Particularly, we built upon our first-hand experience gathered during the mBrain study and utilized two public real-world datasets to illustrate both the challenges and the proposed countermeasures. This way, our work aimed to practically address the overlooked challenges in data collection and retrospective analysis in ambulatory wearable monitoring studies.

Regarding the participant- and data-entry-related challenges, a key overarching conclusion is that any component requiring user interactions should be intricately tied to the research objective and demand minimal user effort^13,20^. The selected wearable device should align with the research goal in terms of measuring sensitivity and user burden, with minimizing user burden being paramount in longitudinal research settings^21^. Participant compliance can be monitored via compliance visualizations leveraging near real-time participant data streams, enabling timely re-instruction. To mitigate implicitness assumptions and minimize the likelihood of data entry errors, it is advisable to conduct monitoring studies in incremental waves, starting with a pilot study. Questionnaires can help address implicitness assumptions, and incorporating tailored questionnaires that gauge for context can aid in assessing personal bias for highly subjective event labels like stress.

Turning to wearable-related data quality challenges, visualization plays a critical role in evaluating the quality of different signal modalities during data processing and analysis steps. Tools like tsflex and Plotly-Resampler enhance the ability to process and visualize these data modalities efficiently and at scale. We introduced an algorithm that performs better in both inference speed and accuracy for identifying non-wear periods, developed using these toolkits. A non-wear detection pipeline is essential to filter out non-wear bouts before further processing and analysis. Finally, we propose a bootstrapping methodology to assess the impact of incorporating incomplete windows-of-interest on analysis metrics.

In conclusion, we present practical solutions to prominent challenges in ambulatory monitoring research, thereby enhancing the quality and efficacy of data collection and analysis. By openly sharing our code scripts and a subset of the mBrain study data, we facilitate reproducibility and enable direct applicability in real-world settings.

### Supplementary Information

Supplementary Information.

## Data Availability

All code and a patient sample of the mBrain study are publicly available at https://github.com/predict-idlab/data-quality-challenges-wearables and https://www.kaggle.com/datasets/jonvdrdo/mbrain21/data, respectively.
