# Supplementary material for: Mitigating data quality challenges in ambulatory wrist-worn wearable monitoring through analytical and practical approaches
Source: Sci Rep. 2024 Jul 30;14:17545. doi: 10.1038/s41598-024-67767-3 (PMC11289092; doi:10.1038/s41598-024-67767-3)
Supplement: Supplementary file 1 — Supplementary Information. [file 41598_2024_67767_MOESM1_ESM.pdf]

Supplementary Materials

For

Mitigating Data Quality Challenges in Ambulatory Wrist-worn Wearable Monitoring Through Analytical and Practical Approaches

Jonas Van Der Donckt, Nicolas Vandenbussche, Jeroen Van Der Donckt, Stephanie Chen , Marija Stojchevska, Mathias De Brouwer, Bram Steenwinckel, Koen Paemeleire, Femke Ongenae, Sofie Van Hoecke

Open Science Statement

All data and corresponding code are openly available through Github and Kaggle datasets.

Github: https://github.com/predict-idlab/data-quality-challenges-wearables
Kaggle datasets: https://www.kaggle.com/datasets/jonvdrdo/mbrain21/data

Contents

S1: mBrain21 application screenshots **2**
S2: Participant compliance report of the ETRI lifelog 2020 study **3**
S3: Non-wear Detection: Algorithm Comparison **4**
S4: Comparison of Gap Induction Procedures **6**

S1: mBrain21 application screenshots

Supplemental Figure 1: mBrain application timeline of a dummy participant, showcasing contextual data, including user-defined semantic locations (e.g. “Work”).

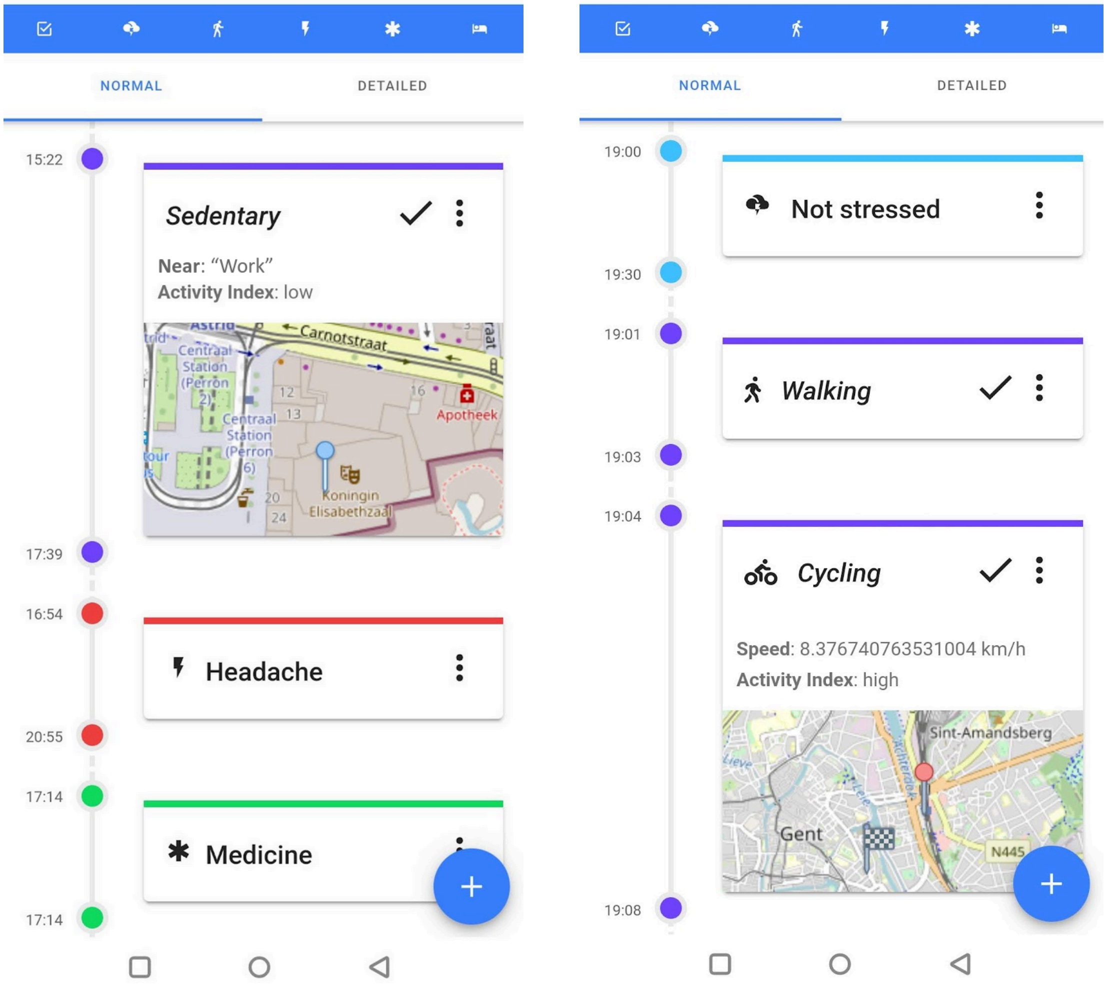

(a) (b)

Note. The streamed data of the Empatica E4 device is utilized to generate activity and stress timeline predictions. Unconfirmed predictions can be validated by interacting with the checkmark (✓) symbol.

S2: Participant compliance report of the ETRI lifelog 2020 study

Supplemental Figure 2: ETRI lifelog 2020 study interaction visualization of a single participant for a period of 28 days.

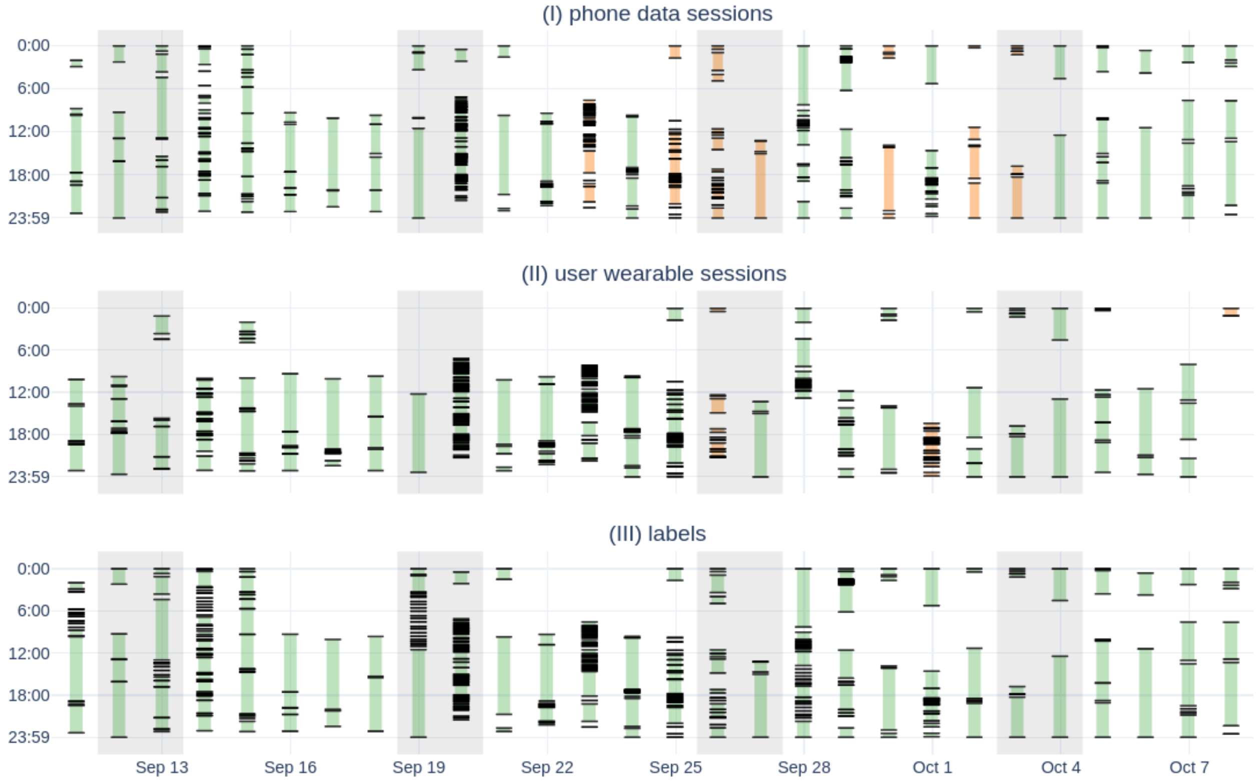

Note. Similar to the mBrain interaction plot (Figure 2), the ETRI interaction visualization utilizes stacked bars to depict phone (i) and wearable (ii) data sessions. Accommodating the fact that participants manually labeled intervals, the label subplot (iii) also uses a bar interval representation, indicating periods for which social and affective labels are present. Remark how the phone and wearable session are a subset of the label session data. In alignment with Figure 2**,** session bars are color-coded in orange when fewer than 8 hours of data are available for the corresponding day.

S3: Non-wear Detection: Algorithm Comparison

This appendix assesses the performance of our revised non-wear detection algorithm relative to the on-body algorithm of Böttcher et al. via metric-based performance evaluation using a labeled subset of the mBrain data. In order to validate on the mBrain dataset, we first created an annotation dashboard to label the mBrain data retrospectively by a single annotator, as depicted in Supplemental Figure 3**.** This allows determining performance assessment metrics, which are shown in Table 1 and Table 2, for ours and Böttcher's algorithm respectively**.** These metrics indicate that our revised algorithm outperforms Böttcher's in both precision and recall, consequently yielding a higher F1-score. Additional data and code specifics can be found in the accompanying notebook.

Supplemental Figure 3: Screenshot of the annotation dashboard utilized to label off-wrist periods.

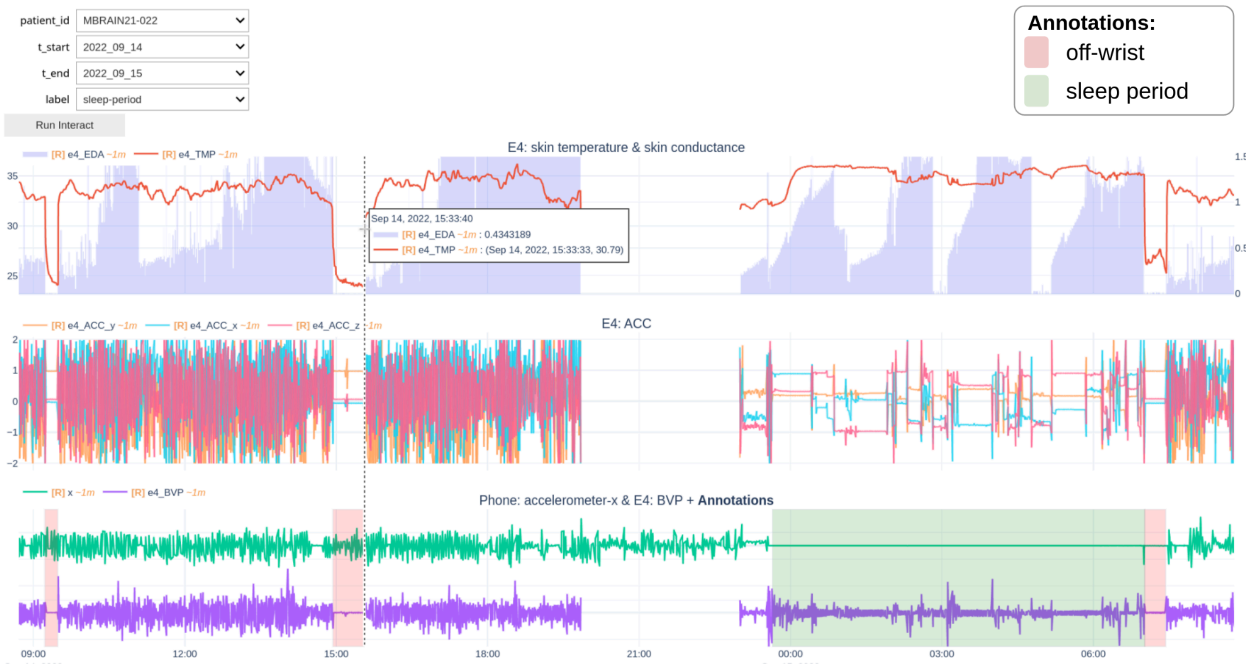

Note: Via the "label" selection box, different labels can be assigned to annotations, each with their own color coding. For the shown excerpt, three off-wrist periods (red shaded area) and one sleep period (green shaded area) were annotated. The code for the annotation dashboard can be found here.

Supplemental Table 1: Classification report of our non-wear detection algorithm.

	precision	recall	f1-score
**Non-wear**	0.89	0.98	0.93
**on-body**	0.98	0.88	0.94
**Macro avg**	0.94	0.94	0.94

Supplemental Table 2: Classification report of Böttcher’s non-wear detection algorithm.

	precision	recall	f1-score
**Non-wear**	0.45	1	0.66
**on-body**	1	0.65	0.79
**Macro avg**	0.73	0.83	0.71

S4: Comparison of Gap Induction Procedures

In Supplemental figure XX, we present a visual comparison of the effects of various gap induction techniques used during bootstrapping. It is evident from the figure that the variability in sampling-based bootstrapping is considerably less than that in block-based bootstrapping (i.e., range mask and range mask 2). This highly reduced variability suggests that sampling-based bootstrapping may not be suitable for assessing metric-gap sensitivity in wearable data. Interestingly, the difference between range mask and range mask 2 is minimal, suggesting that introducing multiple blocks versus a single large block does not notably alter the variability. Implementation details can be found in this notebook.

Supplemental Figure 4: Strip-plot comparison of gap induction procedures (y-axis) for various retention ratios (hue), metrics (columns), and reference series (rows).

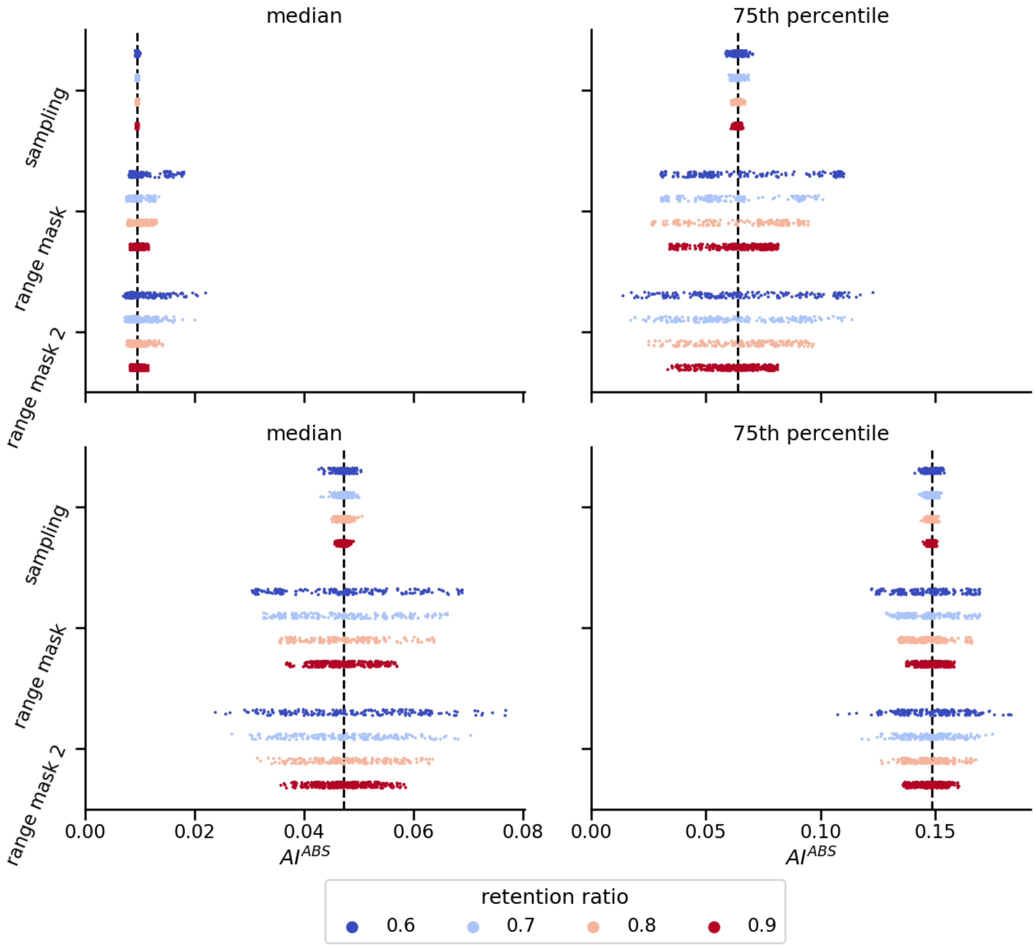

Note: Each row in the figure utilizes a distinct reference series. Columns represent different metrics (i.e., 50th and 75th percentiles). The vertical dashed black line indicates the metric value of the gap-free reference series. This visualization was derived by converting the E4 accelerometer data into a second-by-second activity index, AI^{ABS}, following the methodology of Bai et al. (2016). Data retention ratios during gap induction are differentiated by hue. The y-axis labels the used bootstrapping technique: 'sampling' involves random sample removal; 'range-mask' introduces single block-based gaps; and 'range mask 2' introduces multiple block-based gaps.
